# Dexpanthenol Attenuates Methotrexate‐Induced Nephrotoxicity Through Modulation of NF‐κB–Mediated Inflammation and SIRT1/PGC‐1α–NRF2/HO‐1–Associated Oxidative Stress Pathways

**DOI:** 10.1002/jbt.71055

**Published:** 2026-08-03

**Authors:** Atila Altuntas, Halil Asci, Esma Selcuk, Huzeyfe Karaosman, Osman Aydin, Ilter Ilhan, Sefa Alperen Ozturk, Ozlem Ozmen

**Affiliations:** ^1^ Division of Nephrology, Department of Internal Medicine, Faculty of Medicine Suleyman Demirel University Isparta Türkiye; ^2^ Department of Medical Pharmacology, Faculty of Medicine Suleyman Demirel University Isparta Türkiye; ^3^ Department of Medical Biology, Faculty of Medicine Institute of Health Sciences Isparta Türkiye; ^4^ Department of Internal Medicine, Faculty of Medicine Suleyman Demirel University Isparta Türkiye; ^5^ Department of Internal Medicine Konya State Hospital Konya Türkiye; ^6^ Department of Biochemistry, Faculty of Medicine Suleyman Demirel University Isparta Türkiye; ^7^ Department of Urology, Faculty of Medicine Suleyman Demirel University Isparta Türkiye; ^8^ Department of Pathology, Faculty of Veterinary Medicine Burdur Mehmet Akif Ersoy University Burdur Türkiye

**Keywords:** apoptosis, dexpanthenol, inflammation, Methotrexate, nephrotoxicity, oxidative stress

## Abstract

Methotrexate (MTX)‐induced nephrotoxicity remains a clinically relevant limitation associated with oxidative stress, inflammation, and apoptosis. Dexpanthenol (DEX), a pantothenic acid derivative, has demonstrated cytoprotective properties; however, its effects on MTX‐induced renal injury and related molecular pathways are not fully elucidated. This study investigated the potential renoprotective effects of DEX with a focus on inflammation‐ and redox‐associated signaling. Thirty‐two male Wistar rats were allocated into four groups: Control, MTX, MTX + DEX, and DEX. Renal injury was assessed by histopathology, immunohistochemical analysis of caspase‐3, NF‐κB, and TNF‐α, biochemical parameters (urea and creatinine), and RT‐qPCR analysis of SIRT1, PGC‐1α, NRF2, and HO‐1 gene expression. MTX administration resulted in marked renal damage characterized by tubular degeneration, hyperemia, and inflammatory infiltration, accompanied by increased caspase‐3, NF‐κB, and TNF‐α expression (*p* < 0.001). MTX also significantly suppressed SIRT1, PGC‐1α, NRF2, and HO‐1 gene expression (*p* < 0.001). DEX co‐treatment attenuated histopathological injury and significantly reduced pro‐inflammatory and apoptotic markers while restoring SIRT1, PGC‐1α, and HO‐1 expression (*p* < 0.01–0.001), with a non‐significant upward trend in NRF2 levels. Biochemically, DEX reduced MTX‐induced urea and creatinine elevation. DEX confers significant protection against MTX‐induced renal injury, likely through modulation of inflammatory and oxidative stress–related regulatory pathways and attenuation of apoptosis. These findings support the potential of DEX as a pharmacological candidate for mitigating drug‐induced nephrotoxicity; however, further studies at the protein and functional levels are warranted.

## Graphical abstract

Graphical abstract depicting the protective role of DEX against MTX‐induced nephrotoxicity.

MTX exposure activates NF‐κB, leading to upregulation of TNF‐α and downstream apoptotic mediator Cas‐3, which collectively drive inflammation, apoptosis, and renal tissue damage. Simultaneously, MTX suppresses protective regulators SIRT1, PGC‐1α, NRF2, and HO‐1, resulting in impaired mitochondrial biogenesis and weakened antioxidant defense. In contrast, DEX co‐treatment inhibits the NF‐κB/TNF‐α/Cas‐3 cascade, thereby reducing inflammatory and apoptotic signaling, while restoring the SIRT1/PGC‐1α/NRF2/HO‐1 pathways, leading to preserved renal architecture, reduced oxidative stress, and improved renal function. The figure summarizes the integrated mechanistic framework underlying the renoprotective action of DEX. MTX: Methotrexate, DEX: Dexpanthenol, SIRT1: Sirtuin 1, PGC‐1α: Peroxisome proliferator‐activated receptor‐gamma coactivator‐1alpha, NRF2: Nuclear factor erythroid 2‐related factor 2, HO‐1: heme oxygenase 1, TNF‐α: Tumor necrosis factor alpha, NF‐κB: Nuclear factor kappa B, Cas‐3: Cas‐3.

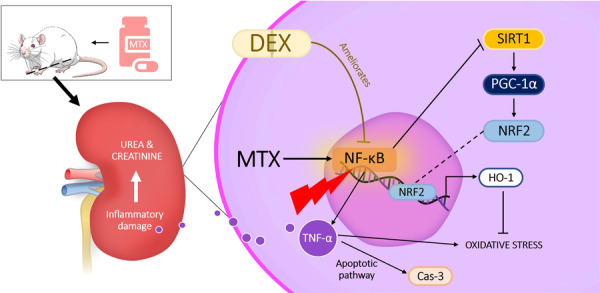

## 1 Introduction

Methotrexate (MTX) is a widely used chemotherapeutic and immunosuppressive agent in the management of malignancies and autoimmune disorders; however, its clinical utility is frequently limited by dose‐dependent nephrotoxicity. The underlying pathogenesis of MTX‐induced renal injury involves a complex interplay of oxidative stress, inflammation, and apoptosis, ultimately leading to structural and functional impairment of renal tissue [1, 2]. Experimental evidence has demonstrated that MTX promotes activation of key pro‐inflammatory and pro‐apoptotic mediators, including nuclear factor kappa B (NF‐κB), tumor necrosis factor‐α (TNF‐α), and caspase‐3 (Cas‐3), which collectively contribute to renal cellular damage [3, 4, 5, 6]. In parallel, MTX disrupts endogenous cytoprotective mechanisms by suppressing critical regulatory pathways associated with mitochondrial function and redox homeostasis, such as the sirtuin 1 (SIRT1)/peroxisome proliferator‐activated receptor gamma coactivator‐1α (PGC‐1α) axis and the nuclear factor erythroid 2–related factor 2 (NRF2)/heme oxygenase‐1 (HO‐1) antioxidant system [5, 7, 8]. These findings suggest that MTX‐induced nephrotoxicity is driven by coordinated dysregulation of interconnected molecular networks rather than a single dominant pathway.

Dexpanthenol (DEX), a biologically active derivative of pantothenic acid, has gained attention for its antioxidant, anti‐inflammatory, and anti‐apoptotic properties. Previous studies have reported that DEX mitigates tissue injury in various experimental models, including sepsis‐associated acute kidney injury, primarily through attenuation of oxidative stress and inflammatory signaling [9, 10, 11]. In addition, emerging evidence suggests that DEX may influence key regulatory pathways involved in cellular stress responses, including those linked to mitochondrial biogenesis and redox balance [12]. Despite these promising findings, the potential protective effects of DEX in MTX‐induced nephrotoxicity—and its impact on the coordinated regulation of inflammation‐ and oxidative stress‐related regulatory pathways—remain insufficiently characterized.

Given the multifactorial and interrelated pathophysiology of MTX‐induced renal injury, a comprehensive evaluation of potential therapeutic strategies requires an integrated mechanistic perspective that addresses oxidative stress, inflammation, and cellular defense systems simultaneously. In particular, the dynamic crosstalk between NF‐κB–mediated pro‐inflammatory signaling and the SIRT1/PGC‐1α and NRF2/HO‐1–dependent cytoprotective pathways appears to be a critical determinant of the balance between renal injury progression and tissue recovery. Disruption of this tightly regulated network may amplify inflammatory cascades and exacerbate mitochondrial dysfunction, thereby worsening renal impairment. In this context, biochemical markers of renal function provide indispensable translational evidence linking molecular alterations to functional outcomes, as they reflect the degree of glomerular and tubular injury in experimental models [13, 14].

Although DEX is widely recognized for its topical applications, increasing experimental evidence suggests that its biological effects are not restricted to epithelial tissues. Systemically administered DEX has demonstrated anti‐inflammatory, antioxidant, and cytoprotective properties in several organ injury models, including sepsis‐associated acute kidney injury, hepatic injury, and hypoxic‐ischemic tissue damage. Therefore, evaluating DEX as a potential nephroprotective agent against MTX‐induced renal injury is biologically plausible and may contribute to the repurposing of this clinically available compound. Therefore, the present study aimed to investigate the renoprotective effects of DEX in an experimental model of MTX‐induced kidney injury using a multimodal analytical framework. Histopathological evaluation, immunohistochemical assessment of inflammatory and apoptotic markers (Cas‐3, NF‐κB, TNF‐α), and gene expression analysis of mitochondrial and antioxidant regulators (SIRT1, PGC‐1α, NRF2, HO‐1) were combined to provide an integrated characterization of renal injury and response to treatment. We hypothesized that DEX attenuates MTX‐induced renal damage by modulating inflammation‐ and oxidative stress–related regulatory pathways and reducing apoptotic activity.

## 2 Materials and Methods

### 2.1 Animals and Ethical Approval

A total of 32 adult male Wistar albino rats (weighing between 300 and 350 g) were kept in a controlled environment with stable temperature (21 ± 2°C), humidity (55%–60%), and a 12 h light/dark cycle. The animals had unrestricted access to standard food and water. They were allowed to acclimate to their surroundings for at least 7 days before the commencement of the experiments. Random allocation of the animals was carried out using a random number generator.

This research adhered to the principles outlined in the ARRIVE 2.0 guidelines for reporting animal experiments. All procedures conformed to institutional ethical standards and were approved by the local ethics committee of Süleyman Demirel University (Approval No: 07/568, approval date: 03.07.2025).

### 2.2 Drugs and Chemicals

MTX (500 mg/20 mL injectable solution) was obtained from Koçak Farma, Istanbul, Türkiye. DEX (Bepanthen®, 500 mg/2 mL injectable solution) was supplied by Bayer, Türkiye. Physiological saline (0.9% NaCl) was used as vehicle in control applications.

Ketamine hydrochloride (Keta‐Control®, Doğa İlaç, Türkiye) and xylazine hydrochloride (XylazinBio® 2%, Bioveta PLC, Czech Republic) were used for anesthesia.

Primary antibodies used for immunohistochemical analyses included anti‐Cas‐3 (p12), anti‐NF‐κB (p65), and anti‐TNF‐α antibodies (all from Abcam, Cambridge, UK; dilution 1:100). Biotinylated secondary antibodies and the streptavidin–biotin–peroxidase detection system were used according to the manufacturer's instructions. 3,3′‐diaminobenzidine (DAB) was employed as chromogen.

For RNA isolation, GeneAll RiboEx™ RNA Isolation Kit (GeneAll Biotechnology, Seoul, Korea) was used. cDNA synthesis was performed using A.B.T.™ cDNA Synthesis Kit (Atlas Biotechnology, Türkiye). RT‐qPCR analyses were carried out using 2× SYBR Green Master Mix (Nepenthe, Türkiye) in a CFX96 RT‐qPCR System (Bio‐Rad, California, USA). GAPDH was used as the internal control gene.

All other chemicals and reagents were of analytical grade and obtained from standard commercial suppliers.

### 2.3 Experimental Protocol

In the control group, 1 mL of saline was administered intraperitoneally (i.p.) once daily for seven consecutive days. To ensure procedural consistency, an additional 1 mL of saline was administered i.p. on Day 2.

In the MTX group, a single dose of 20 mg/kg MTX was administered i.p. on Day 2 to induce acute renal injury, while 1 mL of saline was administered i.p. once daily for seven consecutive days [15].

In the MTX + DEX group, rats received 500 mg/kg DEX i.p. once daily for seven consecutive days (Day 1–Day 7). In addition, a single dose of 20 mg/kg MTX was administered i.p. on Day 2 [16].

MTX was administered on Day 2 to allow 1 day of DEX pre‐treatment before induction of renal injury. This design enabled evaluation of the protective effects of DEX both prior to and following MTX exposure. Previous experimental studies have demonstrated that a single high dose of MTX induces significant biochemical and histopathological alterations within 3–7 days, representing an acute model of nephrotoxicity [17, 18]. Therefore, administering MTX on Day 2 within a 7‐day experimental protocol provided sufficient time for the development of early renal injury while allowing assessment of the modulatory effects of DEX during both the initiation and progression phases of MTX‐induced damage.

All rats were anesthetized with 90 mg/kg ketamine and 8–10 mg/kg xylazine 2 h after the final drug administration [19, 20]. Following a midline abdominal incision, the animals were euthanized by surgical exsanguination via blood collection from the inferior vena cava. Kidneys were immediately excised. One half of each kidney was stored at −20°C for biochemical and gene expression analyses, while the remaining half was fixed in 10% neutral‐buffered formalin for histopathological (H&E) and immunohistochemical evaluation.

### 2.4 Histopathological Examination

Kidney tissues were immersed in 10% neutral‐buffered formalin for a minimum of 48 h to ensure optimal preservation of tissue architecture. Subsequently, samples were processed using a fully automated tissue processor (Leica ASP300S) and embedded in paraffin blocks. Serial sections of 5 µm thickness were obtained using a rotary microtome (Leica RM2155), carefully mounted onto glass slides, and stained with H&E for detailed histological evaluation under light microscopy.

Histopathological examination was conducted independently by an experienced pathologist who was blinded to the experimental groups in order to minimize bias. A standardized semi‐quantitative scoring system was employed, assessing key pathological parameters including tubular epithelial degeneration and necrosis, interstitial hemorrhage, inflammatory cell infiltration, and hyperemia. Each parameter was graded on a scale ranging from 0 to 3 (0 = absent, 1 = mild, 2 = moderate, 3 = severe), allowing for systematic and reproducible evaluation.

For each specimen, at least ten randomly selected non‐overlapping high‐power fields (40× magnification) were examined, and mean scores were calculated for each pathological feature. These data provided a quantitative basis for intergroup comparisons, enabling robust assessment of renal injury severity.

### 2.5 Immunohistochemical Examination

Three serial sections were obtained from each paraffin‐embedded kidney block and mounted on poly‐L‐lysine coated glass slides to enhance tissue adhesion. Immunohistochemical staining was performed using the streptavidin–biotin–peroxidase method to evaluate the expression of Cas‐3, NF‐κB, and TNF‐α. Briefly, sections were deparaffinized in xylene, rehydrated through graded ethanol series, and subjected to heat‐induced antigen retrieval in citrate buffer (pH 6.0). Endogenous peroxidase activity was quenched with 3% hydrogen peroxide for 10 min at room temperature. Slides were then incubated with primary antibodies (Anti‐Cas‐3 p12, Anti‐NF‐κB p65, Anti‐TNF‐α; all diluted) for 60 min at room temperature in a humidified chamber. After washing, sections were treated with biotinylated secondary antibodies, followed by streptavidin–alkaline phosphatase complexes. Immunoreactivity was visualized with DAB chromogen, and sections were counterstained with hematoxylin, dehydrated, and coverslipped for microscopic evaluation. Negative control slides were processed in parallel with the omission of primary antibodies to confirm staining specificity.

Immunohistochemical evaluation was carried out independently by a pathologist blinded to the treatment groups. For each section, ten randomly selected non‐overlapping high‐power fields (40×) were analyzed. Color deconvolution was applied using the DAB‐Hematoxylin vector in ImageJ (v1.48, NIH, Bethesda, MD, USA), A constant threshold value was used for all images, and the percentage of positively stained area was calculated. While photomicrographs and morphometric measurements were documented with the CellSens Imaging System (Olympus, Tokyo, Japan). Quantitative data were systematically recorded, and mean values were calculated for each experimental group. Statistical analyses were subsequently conducted to compare expression levels across groups, enabling objective assessment of the immunohistochemical alterations in relation to experimental interventions.

### 2.6 RT‐qPCR Examination

Using the manufacturer's protocol, RNA was isolated from homogenized tissues with the GeneAll RiboEx (TM) RNA Isolation Kit. The amount and purity of the RNAs obtained were measured with the BioSpec‐nano nanodrop (Shimadzu Ltd. Kyoto, Japan) device. A total of 1 µg of RNA was used for cDNA synthesis. cDNA synthesis was carried out using the A.B.T.™ cDNA Synthesis Kit according to the manufacturer's protocol. Primer designs were made by detecting specific mRNA sequences and testing possible primer sequences using the NCBI website. The sequences of the primer sequences used are shown in Table 1. Expression levels of genes were measured in a Biorad CFX96 real‐time PCR instrument using 2X SYBR green master mix. In the study, the GAPDH gene was used as a housekeeping gene. The reaction mixture was prepared according to the manufacturer's protocol to a final volume of 20 µl. The resulting reaction mixture was placed in a real‐time qPCR device determined according to the kit manufacturer's protocol, and each sample was studied in 3 replications. PCR conditions, initial denaturation 94°C 10 min. 1 cycle, denaturation 95°C 15 s. and annealing/extension 55°C 30 s. was applied as 40 cycles. Relative mRNA levels were calculated by applying the ^2^(‐ΔΔCt) method to the normalized results. Each biological sample represented an individual animal, and all RT‐qPCR reactions were performed in triplicate technical replicates.

**Table 1:** Primer sequences, product size and accession numbers of genes.

Genes	Primer sequence	Product size	Accession number
GAPDH (houseKeeping)	F: AGTGCCAGCCTCGTCTCATA	248 bp	NM_017008.4
	R: GATGGTGATGGGTTTCCCGT		
SIRT1	F: GGTAGTTCCTCGGTGTCCT	152 bp	NM_001414959.1
	R: ACCCAATAACAATGAGGAGGTC		
PGC‐1α	F: TTCAGGAGCTGGATGGCTTG	104 bp	NM_031347.1
	R: AGATCTGGGCAAAGAGGCTG		
NRF2	F: GCCTTCCTCTGCTGCCATTAGTC	126 bp	NM_001399173.1
	R: TCATTGAACTCCACCGTGCCTTC		
HO‐1	F: AGCCTGGTTCAAGATACTACCTC	240 bp	XM_039097470.1
	R: AGGCCCAAGAAAAGAGAGCC		

Abbreviations: F, Forward; GAPDH, Glyceraldehyde‐3‐phosphate dehydrogenase; HO‐1, Heme oxygenase‐1; NRF2, Nuclear factor erythroid 2‐related factor 2; PGC‐1α, Peroxisome proliferator‐activated receptor‐gamma coactivator‐1alpha; R, Reverse; SIRT1, Sirtuin 1.

### 2.7 Biochemical Examination

Blood samples collected from the rats were transferred into gel‐containing tubes. Samples were then centrifuged at 6.000 RPM for 10 min at 4°C to obtain serum. The separated serum samples were stored at −20°C until biochemical analyses were performed.

Serum creatinine (mg/dL) and urea (mg/dL) levels were quantified using a spectrophotometric technique with the Beckman Coulter AU 5800 automated analyzer (Beckman Coulter, USA).

### 2.8 Statistical Analysis

All statistical analyses were performed using GraphPad Prism software (version 10.1.0; GraphPad Software, San Diego, CA, USA). Data distribution was assessed for normality using the Shapiro–Wilk test. As all datasets demonstrated normal distribution (*p* > 0.05), parametric tests were applied.

Comparisons among experimental groups (Control, MTX, MTX + DEX, and DEX) were performed using one‐way analysis of variance (ANOVA) for histopathological scores, immunohistochemical data, gene expression levels, and biochemical parameters. When a significant overall effect was observed, Tukey's multiple comparison post hoc test was conducted to determine pairwise differences between groups. Results are presented as mean ± standard deviation (SD). A *p*‐value of < 0.05 was considered statistically significant.

A priori power analysis was conducted using G*Power software (version 3.1.9.7, Universität Düsseldorf, Germany) to determine the minimum required sample size. The analysis was based on an effect size (f) of 0.40, an alpha level of 0.05, and a statistical power (1–β) of 0.80, indicating that the sample size (*n* = 8 per group) was sufficient to detect statistically meaningful differences among groups.

## 3 Results

### 3.1 Histopathological Findings

MTX administration induced severe renal injury characterized by pronounced hyperemia, interstitial hemorrhage, tubular degeneration, and necrosis. Tubular epithelial cells exhibited cytoplasmic vacuolization, nuclear pyknosis, and luminal desquamation, frequently accompanied by proteinaceous casts. Marked mononuclear inflammatory cell infiltration was also observed.

**Figure 1:**
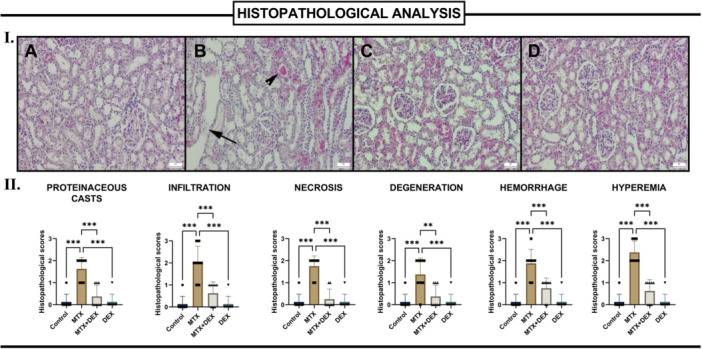
Histopathological alterations in renal tissues following MTX administration and the protective effects of DEX. I: Representative histopathological appearance of kidneys between the groups. (A) Normal renal architecture in the Control group; (B) Severe histopathological pathological alterations, including hyperemia, interstitial hemorrhage, inflammatory cell infiltration (arrow), tubular degeneration, necrosis, and proteinaceous casts (arrow head) in the MTX group; (C) Markedly attenuated tissue injury in the MTX + DEX group; (D) Normal kidney histology in the DEX group, HE staining, Scale bars = 50 µm. II: Statistical analysis results of the groups. Data are presented as mean ± SD. Statistical analysis was performed by Kruskal‐Wallis followed by Dunn test. ***p < 0.001, **p < 0.01. MTX: Methotrexate, DEX: Dexpanthenol.

Co‐treatment with DEX markedly attenuated these alterations, with preservation of tubular architecture and substantial reduction in vascular changes and inflammatory infiltration. In contrast, the control and DEX‐only groups displayed normal renal histoarchitecture without detectable pathological changes (Figure 1).

### 3.2 Immunohistochemical Findings

#### 3.2.1 TNF‐α Immunohistochemistry

**Figure 2:**
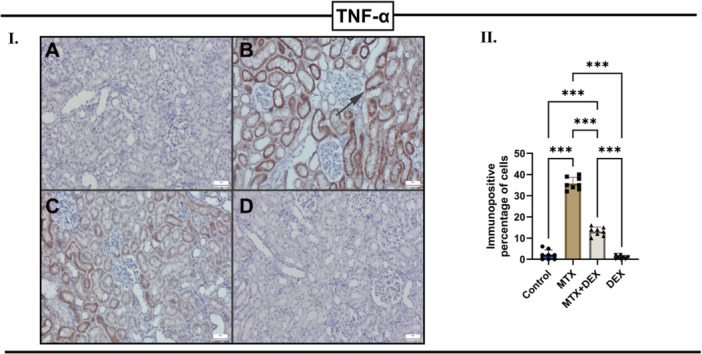
Immunohistochemical expression of TNF‐α in kidneys between the experimental groups. I: Representative micrographs, (A) Negative to minimal TNF‐α immunoreactivity in the Control group; (B) Marked TNF‐α expression in tubular epithelial cells (arrow), in the MTX group; (C) Markedly reduced immunostaining in the MTX + DEX group; (D) Negative expressions in the DEX group, streptavidin–biotin–peroxidase method, scale bars = 50 µm. II: Statistical analysis results of the groups. Data are presented as mean ± SD. Analysis was performed by Kruskal‐Wallis followed by Dunn test. ***p < 0.001. MTX: Methotrexate, DEX: Dexpanthenol, TNF‐α: Tumor necrosis factor alpha.

MTX significantly increased TNF‐α immunoreactivity compared with the control group (*p* < 0.001), with strong cytoplasmic staining predominantly in tubular epithelial cells. DEX co‐treatment significantly reduced TNF‐α expression relative to MTX alone (*p* < 0.001). Control and DEX groups showed minimal staining (Figure 2).

#### 3.2.2 NF‐κB Immunohistochemistry

**Figure 3:**
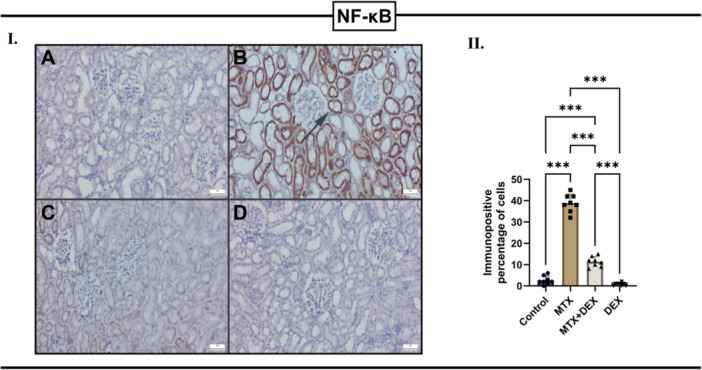
Immunohistochemical expression of NF‐κB in kidneys between the experimental groups. I: Representative micrographs, (A) Negative expressions in the Control group; (B) Markedly increased NF‐κB expression in tubular epithelial cells (arrow) in the MTX group; (C) Decreased NF‐κB expressions in the MTX + DEX group; (D) Negative expression in the DEX group, streptavidin–biotin–peroxidase method, scale bars = 50 µm. II: Statistical analysis results of the groups. Data are shown as mean ± SD. Analysis was performed by Kruskal‐Wallis followed by Dunn test. ***p < 0.001. MTX: Methotrexate, DEX: Dexpanthenol, NF‐κB: Nuclear factor kappa B.

NF‐κB expression was markedly elevated in the MTX group (*p* < 0.001 vs. control). This increase was significantly attenuated by DEX treatment (*p* < 0.001 vs. MTX). Baseline expression levels were observed in the control and DEX groups (Figure 3).

#### 3.2.3 Cas‐3 Immunohistochemistry

**Figure 4:**
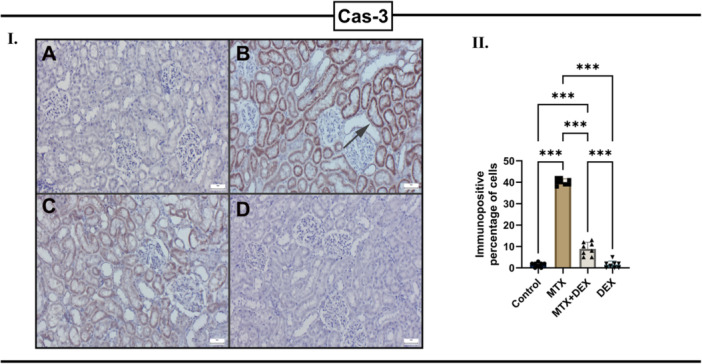
Immunohistochemical expression of Cas‐3 in kidneys between the experimental groups. I: Representative micrographs, (A) Negative expressions in the Control group; (B) Markedly increased Cas‐3 expression in tubular epithelial cells (arrow) in the MTX group; (C) Decreased Cas‐3 expression in the MTX + DEX group; (D) Negative expression in the DEX group, streptavidin–biotin–peroxidase method, scale bars = 50 µm. II: Statistical analysis results of the groups. Data are shown as mean ± SD. Statistical analysis was performed by Kruskal‐Wallis followed by Dunn test. ***p < 0.001. MTX: Methotrexate, DEX: Dexpanthenol, Cas‐3: Caspase‐3.

Caspase‐3 expression was significantly increased following MTX administration (*p* < 0.001 vs. control), indicating enhanced apoptotic activity. DEX significantly reduced Cas‐3 immunoreactivity compared with the MTX group (*p* < 0.001), whereas control and DEX‐only groups exhibited minimal staining (Figure 4).

#### 3.2.4 Genetic Findings

**Figure 5:**
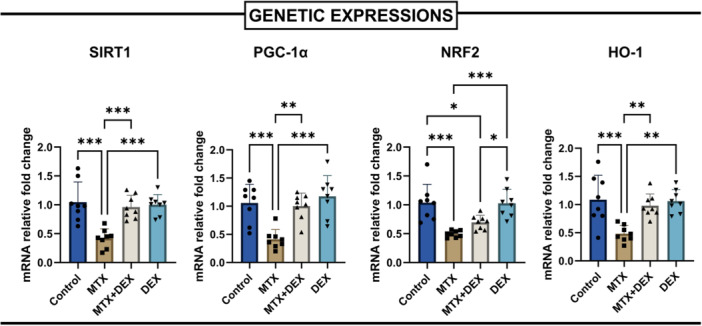
Statistical analysis results of SIRT1, PGC‐1α, NRF2, and HO‐1 renal gene expression between the groups. RT‐qPCR analysis demonstrated significant downregulation of SIRT1, PGC‐1α, NRF2, and HO‐1 in the MTX group compared to controls (*p* < 0.001). Co‐administration of DEX (MTX + DEX group) significantly increased the expression of SIRT1, PGC‐1α, and HO‐1 compared with the MTX group (*p* < 0.01–0.001), whereas NRF2 expression showed an increasing trend that did not reach statistical significance. The DEX‐only group exhibited gene expression levels comparable to control, confirming the absence of adverse effects under physiological conditions. Data are presented as mean ± SD. Analysis was performed by Kruskal‐Wallis followed by Dunn test. ***p < 0.001, **p < 0.01, *p < 0.05. MTX: Methotrexate, DEX: Dexpanthenol, SIRT1: Sirtuin 1, PGC‐1α: Peroxisome proliferator‐activated receptor‐gamma coactivator‐1alpha, NRF2: Nuclear factor erythroid 2‐related factor 2, HO‐1: Heme oxygenase‐1.

MTX exposure resulted in significant downregulation of SIRT1, PGC‐1α, NRF2, and HO‐1 expression compared with controls (*p* < 0.001). DEX co‐treatment significantly restored SIRT1, PGC‐1α, and HO‐1 expression levels (*p* < 0.01–0.001 vs. MTX), while NRF2 expression showed an increasing trend that did not reach statistical significance. Gene expression levels in the DEX‐only group were comparable to control (Figure 5).

## 4 Discussion

**Figure 6:**
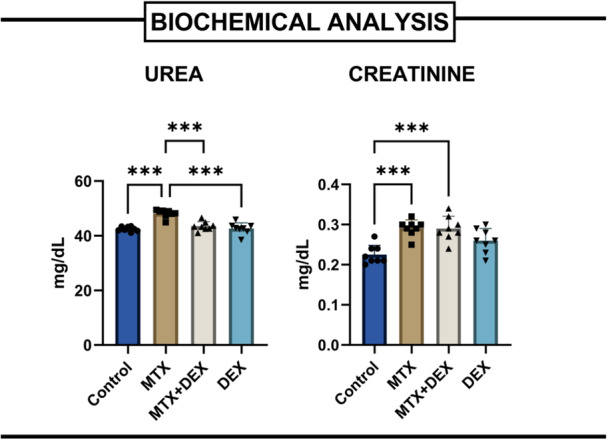
Statistical analysis results of biochemical analysis of serum urea and creatinine levels in experimental groups (*n* = 8 per group). MTX administration significantly increased both urea and creatinine levels compared with the Control group (*p* < 0.001). Co‐treatment with DEX (MTX + DEX) markedly reduced urea elevation compared with MTX alone (*p* < 0.001), while values in the DEX group were comparable to controls. Data are presented as mean ± SD. Statistical analysis was performed by one‐way ANOVA followed by Tukey test. ***p < 0.001. MTX: Methotrexate, DEX: Dexpanthenol.

The present study demonstrates that MTX induces pronounced renal injury characterized by structural disruption, inflammatory activation, and apoptotic signaling, while DEX confers significant protection across histopathological, biochemical, and molecular domains. Importantly, our findings provide integrated evidence that the renoprotective effects of DEX are associated with attenuation of inflammatory and apoptotic responses together with restoration of redox‐ and mitochondria‐related regulatory pathways. The selected molecular panel was intentionally designed to evaluate interconnected inflammatory and cytoprotective pathways rather than a single isolated mechanism. NF‐κB/TNF‐α signaling, apoptosis‐related Cas‐3 activation, and SIRT1/PGC‐1α–NRF2/HO‐1‐associated antioxidant responses constitute a biologically integrated network that collectively regulates MTX‐induced renal injury (Figure 6).

MTX‐induced renal injury observed in this study was marked by severe tubular degeneration, hyperemia, and inflammatory cell infiltration. These findings are consistent with previous reports identifying oxidative stress and inflammation as central drivers of MTX nephrotoxicity [1, 2]. The marked increase in NF‐κB and TNF‐α expression further supports the role of inflammatory signaling in mediating endothelial activation and leukocyte recruitment [21]. In parallel, elevated caspase‐3 immunoreactivity indicates activation of apoptosis, which is a well‐established downstream consequence of oxidative and inflammatory injury [22]. Together, these data confirm that MTX‐induced nephrotoxicity involves coordinated activation of inflammatory and apoptotic pathways.

DEX treatment markedly attenuated these pathological alterations. The reduction in NF‐κB and TNF‐α expression suggests suppression of pro‐inflammatory signaling, while decreased caspase‐3 immunoreactivity indicates mitigation of apoptosis. These findings are in agreement with previous studies demonstrating the anti‐inflammatory and anti‐apoptotic properties of DEX in experimental injury models [10, 11]. Notably, the concurrent modulation of both inflammatory and apoptotic markers suggests that DEX exerts a broad cytoprotective effect rather than acting on a single downstream target.

At the molecular level, MTX significantly suppressed the expression of SIRT1, PGC‐1α, NRF2, and HO‐1, indicating disruption of mitochondrial function and antioxidant defense systems. These pathways are known to play critical roles in maintaining cellular energy homeostasis and redox balance under stress conditions [23, 24]. In contrast, DEX co‐treatment restored SIRT1, PGC‐1α, and HO‐1 expression and partially increased NRF2 levels. Although NRF2 upregulation did not reach statistical significance, the overall expression pattern suggests a trend toward reactivation of antioxidant signaling. Given the known interaction between SIRT1 and NF‐κB signaling, whereby SIRT1 negatively regulates NF‐κB activity through deacetylation of its p65 subunit [25, 26, 27, 28], the observed changes may reflect coordinated modulation of interconnected pathways rather than isolated effects. Although classical oxidative stress biomarkers such as MDA, GSH, SOD, and CAT were not evaluated, the observed modulation of NRF2, HO‐1, SIRT1, and PGC‐1α suggests an indirect influence of DEX on redox‐regulatory pathways.

Biochemically, MTX administration resulted in significant elevations in serum urea and creatinine levels, indicating impaired renal function. DEX treatment significantly reduced urea levels; however, the decrease in creatinine levels did not reach statistical significance. This discrepancy may be related to the relatively limited sensitivity of creatinine as an early marker of acute kidney injury and the short duration of the experimental protocol. Accordingly, the substantial improvements observed in histopathological alterations and molecular markers suggest that the protective effects of DEX may precede detectable changes in conventional renal functional markers. Nevertheless, the absence of a significant improvement in serum creatinine levels indicates that DEX did not completely reverse MTX‐induced renal dysfunction. This observation further supports the multifactorial nature of MTX nephrotoxicity, which involves several pathogenic mechanisms beyond the inflammatory and oxidative stress–related pathways examined in the present study. Therefore, although DEX effectively attenuated tissue injury and modulated key inflammatory, apoptotic, and redox‐regulatory pathways, its renoprotective effects should be interpreted as partial rather than complete under the experimental conditions employed. The present study has several strengths, including the use of a multimodal analytical approach integrating histopathological, immunohistochemical, biochemical, and gene expression data. This comprehensive design allows for a more holistic evaluation of renal injury and therapeutic response, providing converging evidence across structural, molecular, and functional domains.

It should be emphasized that methotrexate‐induced nephrotoxicity is a multifactorial process. In addition to inflammation, oxidative stress, and apoptosis, renal injury may arise from intratubular methotrexate crystal precipitation, direct tubular epithelial toxicity, mitochondrial dysfunction, altered renal microcirculation, and impairment of tubular transport systems. Among these mechanisms, crystal nephropathy is particularly relevant in high‐dose methotrexate exposure, where precipitation of methotrexate and its metabolites within renal tubules can contribute to tubular obstruction and subsequent renal dysfunction. The present study specifically focused on inflammatory, apoptotic, and redox‐regulatory pathways and was not designed to evaluate crystal formation or other physicochemical mechanisms of injury. Therefore, the observed renoprotective effects of dexpanthenol should be interpreted within the context of the investigated molecular pathways, and its potential influence on crystal‐associated nephrotoxicity remains to be elucidated in future studies.

Nevertheless, several limitations of the present study should be acknowledged. First, although gene expression and immunohistochemical findings suggest modulation of key signaling pathways, the absence of protein‐level validation (e.g., Western blot analysis) limits definitive mechanistic interpretation. Second, the use of a single dose and single experimental time point precludes assessment of dose–response relationships as well as the temporal dynamics of injury progression and recovery. Moreover, NRF2 activity is largely regulated through post‐translational mechanisms, particularly nuclear translocation and protein stabilization; therefore, mRNA expression alone may not accurately reflect its functional activation status. Future studies incorporating nuclear and cytoplasmic protein fractionation together with Western blot analyses would provide more robust mechanistic insight.

Another important limitation is that crystal nephropathy and other non‐inflammatory mechanisms implicated in MTX‐induced renal injury were not evaluated. MTX nephrotoxicity is a multifactorial process, and the pathways investigated in the present study represent only part of its complex pathogenesis. Consistent with this notion, serum creatinine levels were not significantly normalized despite marked improvements in histopathological and molecular parameters. This finding suggests that certain pathogenic mechanisms may persist despite DEX treatment and indicates that its renoprotective effects are likely partial rather than complete under the conditions of the present study. Finally, the exclusive use of a single sex and species may limit the broader generalizability of the findings, highlighting the need for validation in both sexes and additional experimental models.

Future studies incorporating protein‐level analyses, functional assays, and mechanistic interventions across diverse and clinically relevant experimental models are warranted to further validate and extend these findings. From a translational perspective, DEX is a clinically available and well‐tolerated compound, which enhances the potential relevance of the present results. Its apparent ability to modulate multiple injury‐related pathways suggests that DEX may represent a promising adjunctive strategy for mitigating drug‐induced nephrotoxicity. Nevertheless, additional studies are required to confirm these effects at the protein and functional levels and to further elucidate the underlying molecular mechanisms in clinically relevant settings.

## 5 Conclusion

In summary, DEX attenuates MTX‐induced renal injury and was associated with reduced inflammatory and apoptotic responses together with modulation of oxidative stress–related regulatory pathways. However, the lack of complete recovery in renal functional parameters indicates that the protective effects were partial and may not encompass all mechanisms involved in MTX‐induced nephrotoxicity. These findings support the potential of DEX as an adjunctive renoprotective strategy, while further studies are required to determine its effects on additional pathogenic mechanisms, including crystal‐associated renal injury, and to validate these findings at protein and functional levels.

## Author Contributions

**Atila Altuntas:** conceptualization, validation. **Halil Asci:** conceptualization, investigation, funding acquisition, writing – original draft. **Esma Selcuk:** investigation, formal analysis. **Huzeyfe Karaosman:** investigation, writing – original draft. **Osman Aydin:** writing – original draft, software. **Ilter Ilhan:** investigation, formal analysis. **Sefa Alperen Ozturk:** investigation, data curation; resources. **Ozlem Ozmen:** conceptualization, investigation, writing – original draft, writing – review and editing, visualization; methodology; data curation; supervision, resources.

## Conflicts of Interest

The authors declare no conflicts of interest.

## Data Availability

The data of the study results are available from the corresponding author upon request.

## References

[1] W. M. Abdel‐Wahab , N. S. Daifalla , and A. E. Essawy , “L‐Methionine Protects Against Nephrotoxicity Induced by Methotrexate Through Modulation of Redox Status and Inflammation,” Redox Report 28, no. 1 (2023): 2270886, 10.1080/13510002.2023.2270886. 37931136 PMC10629423

[2] Ö. Türkoglu , Z. Usta , K. Çavdarlı , S. Garlı , and N. Alp , “L‐Carnitine Attenuates Methotrexate‐Induced Nephrotoxicity by Modulating BAX/BCL2 Apoptotic Signaling and Preserving Podocyte, Endothelial, and Tubular Integrity,” Journal of Applied Toxicology (February 2026), 10.1002/jat.70096. PMC13432696 41692596

[3] A. M. Abdelwahab , H. A. Habib , M. A. Darwish , Y. A. Bin Jardan , and G. H. Heeba , “Modulation of SIRT‐1, NF‐κB/TNF‐α/IL‐6, and ERK/Caspase‐3 by Lutein Mitigates Methotrexate‐Induced Hepatotoxicity,” Pharmaceuticals 18, no. 12 (2025): 1787, 10.3390/ph18121787. 41471276 PMC12736351

[4] N. Vanlangenakker , T. Berghe , D. Krysko , N. Festjens , and P. Vandenabeele , “Molecular Mechanisms and Pathophysiology of Necrotic Cell Death,” Current Molecular Medicine 8, no. 3 (2008): 207–220, 10.2174/156652408784221306. 18473820

[5] E. F. Wasfey , M. Shaaban , M. Essam , et al., “Infliximab Ameliorates Methotrexate‐Induced Nephrotoxicity in Experimental Rat Model: Impact on Oxidative Stress, Mitochondrial Biogenesis, Apoptotic and Autophagic Machineries,” Cell Biochemistry and Biophysics 81, no. 4 (2023): 717–726, 10.1007/s12013-023-01168-7. 37656380 PMC10611839

[6] N. S. Younis , H. S. Elsewedy , T. M. Shehata , and M. E. Mohamed , “Geraniol Averts Methotrexate‐Induced Acute Kidney Injury via Keap1/Nrf2/HO‐1 and MAPK/NF‐κB Pathways,” Current Issues in Molecular Biology 43, no. 3 (2021): 1741–1755, 10.3390/cimb43030123. 34889889 PMC8929074

[7] N. C. Sabry , H. E. Michel , and E. T. Menze , “Repurposing of Erythropoietin as a Neuroprotective Agent Against Methotrexate‐Induced Neurotoxicity in Rats,” Journal of Psychopharmacology 39, no. 2 (2025): 147–163, 10.1177/02698811241295379. 39535118

[8] Y. Zhou , S. Wang , Y. Li , S. Yu , and Y. Zhao , “SIRT1/PGC‐1α Signaling Promotes Mitochondrial Functional Recovery and Reduces Apoptosis After Intracerebral Hemorrhage in Rats,” Frontiers in Molecular Neuroscience 10 (2018): 443, 10.3389/fnmol.2017.00443. 29375306 PMC5767311

[9] Q. Liao , Z. Feng , H. Lin , et al., “Dexmedetomidine Improves Septic Acute Kidney Injury by Inhibiting Inflammation and Oxidative Stress Through the Activation of the Pink1/Park2 Autophagy Pathway,” Renal Failure 47, no. 1 (2025): 2513677, 10.1080/0886022X.2025.2513677. 40485166 PMC12150617

[10] E. S. Özden , H. Aşcı , H. Büyükbayram , et al., “Dexpanthenol Protects Against Lipopolysaccharide‐Induced Acute Kidney Injury by Restoring aquaporin‐2 Levels via Regulation of the Silent Information Regulator 1 Signaling Pathway,” Korean Journal of Anesthesiology 76, no. 5 (2023): 501–509, 10.4097/kja.23207. 37232072 PMC10562075

[11] X. Zhao , S. Zhang , and H. Shao , “Dexpanthenol Attenuates Inflammatory Damage and Apoptosis in Kidney and Liver Tissues of Septic Mice,” Bioengineered 13, no. 5 (2022): 11625–11635, 10.1080/21655979.2022.2070585. 35510377 PMC9275904

[12] N. Üremiş , M. Aslan , E. Taşlidere , and E. Gürel , “Dexpanthenol Exhibits Antiapoptotic and Anti‐Inflammatory Effects Against Nicotine‐Induced Liver Damage by Modulating Bax/Bcl‐xL, Caspase‐3/9, and Akt/NF‐κB Pathways,” Journal of Biochemical and Molecular Toxicology 38, no. 1 (2024): e23622, 10.1002/jbt.23622. 38229321

[13] M. A. I. Alqrad , D. S. El‐Agamy , S. R. M. Ibrahim , et al., “SIRT1/Nrf2/NF‐κB Signaling Mediates Anti‐Inflammatory and Anti‐Apoptotic Activities of Oleanolic Acid in a Mouse Model of Acute Hepatorenal Damage,” Medicina 59, no. 7 (2023): 1351, 10.3390/medicina59071351. 37512162 PMC10383078

[14] S. Gowda , P. B. Desai , S. S. Kulkarni , V. V. Hull , A. A. Math , and S. N. Vernekar , “Markers of Renal Function Tests,” North American Journal of Medical Sciences 2, no. 4 (2010): 170–173. 22624135 PMC3354405

[15] E. Turkmen Samdanci , M. Huz , O. Ozhan , et al., “Cytoprotective Effects of Molsidomine Against Methotrexate‐Induced Hepatotoxicity: An Experimental Rat Study,” Drug Design, Development and Therapy 13 (2018): 13–21, 10.2147/DDDT.S181550. 30587924 PMC6304250

[16] C. Tayman , U. Çakır , A. Kurt , et al., “Evaluation of Beneficial Effects of Dexpanthenol on Hypoxic‐Ischemic Encephalopathy,” Biotechnic and Histochemistry 99, no. 5 (2024): 260–268, 10.1080/10520295.2024.2365231. 38869860

[17] Ş. Yeşilot and M. Özgöçmen , “Nephroprotective Effect of Resveratrol Against Methotrexate ‐Induced Renal Toxicity in Female Rats,” Mehmet Akif Ersoy University Journal of Health Sciences Institute 10, no. 2 (2022): 123–133, 10.24998/maeusabed.1136994.

[18] M. M. Fahmy , H. A. Habib , E. M. Zeidan , Y. A. B. Jardan , and G. H. Heeba , “Alogliptin Mitigates Methotrexate‐Induced Nephrotoxicity in a Rat Model: Antagonizing Oxidative Stress, Inflammation and Apoptosis,” International Journal of Molecular Sciences 26, no. 19 (2025): 9608, 10.3390/ijms26199608. 41096869 PMC12524975

[19] O. Imeci , H. Asci , S. Asci , M. A. Sevuk , H. S. Sarikaya , and O. Ozmen , “Dapagliflozin Mitigates Lipopolysaccharide‐Induced Neuroinflammation Through Potential Involvement of the IL‐17A/GSK3β Signaling Pathway and Modulation of Inflammatory Cytokines,” European Journal of Pharmacology 1003 (2025): 177913, 10.1016/j.ejphar.2025.177913. 40615105

[20] M. Savran , S. E. Akin , H. E. Camas , et al., “Protective Effect of Dapagliflozin on Lipopolysaccharide‐Induced Acute Lung Injury via the SIRT‐1/PGC‐1α Pathway,” Molecular Biology Reports 52, no. 1 (2025): 171, 10.1007/s11033-025-10267-y. 39878908

[21] T. Liu , L. Zhang , D. Joo , and S. C. Sun , “NF‐κB Signaling in Inflammation,” Signal Transduction and Targeted Therapy 2, no. 1 (2017): 17023. 29158945 10.1038/sigtrans.2017.23 PMC5661633

[22] W. A. M. Ghonimi , M. A. Z. Alferah , N. Dahran , and E. S. El‐Shetry , “Hepatic and Renal Toxicity Following the Injection of Copper Oxide Nanoparticles (CuO NPs) in Mature Male Westar Rats: Histochemical and Caspase 3 Immunohistochemical Reactivities,” Environmental Science and Pollution Research 29, no. 54 (2022): 81923–81937, 10.1007/s11356-022-21521-2. 35739448 PMC9605931

[23] A. Loboda , M. Damulewicz , E. Pyza , A. Jozkowicz , and J. Dulak , “Role of Nrf2/HO‐1 System in Development, Oxidative Stress Response and Diseases: An Evolutionarily Conserved Mechanism,” Cellular and Molecular Life Sciences 73, no. 17 (2016): 3221–3247, 10.1007/s00018-016-2223-0. 27100828 PMC4967105

[24] S. M. Radwan , M. Alqulaly , M. Y. Elsaeed , S. Z. Elshora , A. H. Atwa , and E. F. Wasfey , “L‐Carnitine Reverses Methotrexate‐Induced Nephrotoxicity in Experimental Rat Model: Insight on SIRT1/PGC‐1α/Nrf2/HO‐1 Axis,” Journal of Applied Toxicology 43, no. 11 (2023): 1667–1675, 10.1002/jat.4503. 37312617

[25] S. He , Y. Wang , J. Liu , P. Li , X. Luo , and B. Zhang , “Activating SIRT1 Deacetylates NF‐κB p65 to Alleviate Liver Inflammation and Fibrosis via Inhibiting NLRP3 Pathway in Macrophages,” International Journal of Medical Sciences 20, no. 4 (2023): 505–519, 10.7150/ijms.77955. 37057212 PMC10087625

[26] A. Altuntaş , H. R. Yılmaz , A. Altuntaş , et al., “Caffeic Acid Phenethyl Ester Protects Against Amphotericin B Induced Nephrotoxicity in Rat Model,” BioMed Research International 2014 (2014): 702981, 10.1155/2014/702981. 25032223 PMC4084592

[27] S. D. Chen , D. I. Yang , T. K. Lin , F. Z. Shaw , C. W. Liou , and Y. C. Chuang , “Roles of Oxidative Stress, Apoptosis, PGC‐1α and Mitochondrial Biogenesis in Cerebral Ischemia,” International Journal of Molecular Sciences 12, no. 10 (2011): 7199–7215, 10.3390/ijms12107199. 22072942 PMC3211033

[28] A. A. Mishriki , A. K. Khalifa , D. A. Ibrahim , et al., “Empagliflozin Mitigates Methotrexate‐Induced Nephrotoxicity in Male Albino Rats: Insights on the Crosstalk of AMPK/Nrf2 Signaling Pathway,” Future Journal of Pharmaceutical Sciences 10, no. 1 (2024): 95, 10.1186/s43094-024-00669-3.

